# Biological Effects of Indole-3-Propionic Acid, a Gut Microbiota-Derived Metabolite, and Its Precursor Tryptophan in Mammals’ Health and Disease

**DOI:** 10.3390/ijms23031222

**Published:** 2022-01-22

**Authors:** Piotr Konopelski, Izabella Mogilnicka

**Affiliations:** Laboratory of the Centre for Preclinical Research, Department of Experimental Physiology and Pathophysiology, Medical University of Warsaw, 02-091 Warsaw, Poland; izabellamogilnicka@gmail.com

**Keywords:** gut microbiota, indole-3-propionic acid, oxidative stress, cardiovascular system

## Abstract

Actions of symbiotic gut microbiota are in dynamic balance with the host’s organism to maintain homeostasis. Many different factors have an impact on this relationship, including bacterial metabolites. Several substrates for their synthesis have been established, including tryptophan, an exogenous amino acid. Many biological processes are influenced by the action of tryptophan and its endogenous metabolites, serotonin, and melatonin. Recent research findings also provide evidence that gut bacteria-derived metabolites of tryptophan share the biological effects of their precursor. Thus, this review aims to investigate the biological actions of indole-3-propionic acid (IPA), a gut microbiota-derived metabolite of tryptophan. We searched PUBMED and Google Scholar databases to identify pre-clinical and clinical studies evaluating the impact of IPA on the health and pathophysiology of the immune, nervous, gastrointestinal and cardiovascular system in mammals. IPA exhibits a similar impact on the energetic balance and cardiovascular system to its precursor, tryptophan. Additionally, IPA has a positive impact on a cellular level, by preventing oxidative stress injury, lipoperoxidation and inhibiting synthesis of proinflammatory cytokines. Its synthesis can be diminished in the presence of different risk factors of atherosclerosis. On the other hand, protective factors, such as the introduction of a Mediterranean diet, tend to increase its plasma concentration. IPA seems to be a promising new target, linking gut health with the cardiovascular system.

## 1. Introduction

Over the past two decades, research interest on the interactions between diet, gut microbiota and their host organism has grown. The findings bring new information on the correlation between the activity of symbiotic gut microbiota and the pathophysiology of lifestyle diseases, including obesity [1], diabetes [2] and hypertension [3,4]. Initially, research focused mainly on the role of short-chain fatty acids (SCFAs) [3,5] and carnitine-derived metabolites [6,7,8]. The new data suggest that tryptophan, the essential amino acid, can also be metabolized by microbiota, leading to the synthesis of biologically active group of indoles [9]. So far, research projects on bacterial metabolism of tryptophan focused mainly on actions of indole [10,11], and its liver metabolite, indoxyl sulfate (IS) [12,13]. Indole decreases intestinal inflammation and has positive impact on gastrointestinal and liver homeostasis [10,14]. On the other hand, IS can be classified as uremic toxin, as its concentration increases significantly in chronic kidney disease [9,15]. IS is proposed to be one of the factors linking kidney dysfunction with an increased risk of developing cardiovascular disease [12,16,17].

Until now, the biological actions of indole-3-propionic acid (IPA), another microbiota derived metabolite of tryptophan, have not been properly reviewed in the scientific literature. A review of articles summarizing the impact of tryptophan metabolism on cardiovascular system homeostasis has recently been published, however; the main focus of that paper was kynurenine- and the serotonin pathway [18]. The aim of our study is to provide a comprehensive review of the physiological roles of IPA, and changes in its synthesis in neurological, gastrointestinal and cardiovascular diseases. Scientific papers evaluating the biological effects of this metabolite have multiplied greatly over the past few years [19,20,21,22]. IPA has a protective role on a cellular and tissue level, by limiting inflammation [23], lipid peroxidation [24], and the formation of free radicals [25]. Additionally, IPA affects the function of the immune [22], nervous [26], gastrointestinal [27] and cardiovascular system [19], and its synthesis decreases significantly in several pathogenic states and diseases, including colitis [28], diabetes [29], and obesity [30]. The novelty of this review is that we assess the available evidence on biological effects of IPA and emphasize its potential therapeutic applications.

## 2. Gut Microbiota

The close relationship between host and symbiotic gut microbiota has gained scientific interest over the past few decades. It has been observed that this relationship is bidirectional and symbiotic, with both participants benefiting from this union. The host provides a specific niche, supplies nutrients and optimal conditions for the bacteria to grow. The role of gut microbiota in mammalian health goes beyond the synthesis of vitamins [31] and is associated with many biochemical pathways and the synthesis of specific metabolites that can be absorbed into the circulation [32]. Gut bacteria participate in the breakdown of haemoglobin through their ability to transform bilirubin into stercobilinogen, enabling elimination of the latter in faeces [33]. Additionally, gut bacteria metabolize primary bile acids, synthesized in the liver to form secondary bile acids, demonstrating a protective role in gastrointestinal physiology [34]. 

### 2.1. Gut-Blood Barrier (GBB) and Microbiota

The intestinal lumen forms a specific environment for symbiotic microbiota, enabling its growth and physiological function. Certain factors, including oxygen concentration and composition of nutrients, form specific conditions of this microbial environment. It has been observed that composition and richness of microbiota differ significantly between the small and large intestine [35]. Even within the large intestine some researchers have provided evidence that caecal and colon contents should be analysed separately, due to the significant differences in conditions for growth of symbiotic bacteria [36,37]. In rodents, cecum is proposed to have critical role in bacterial fermentation, however; its localization and distance from the anal margin disturb its evaluation in live subjects [38]. Intercaecal administration of investigated compounds and the collection of samples requires surgical preparation [39,40] and potential use of antibiotics that might significantly affect microbial data. Due to this fact, the colon is more frequently used for the evaluation of microbial communities and their impact on health in mammals [41,42]. The gut lumen is separated from the circulation by layers of tissues, which altogether form the gut-blood barrier (GBB). This barrier is not limited to the epithelium and mucous layers. Gut-associated lymphoid tissue (GALT), symbiotic bacteria and secretory proteins, including lactoferrin, prevent the transmission of pathogenic bacteria and their toxins into circulation [43]. Disruption of this protective action of GBB has been observed in several diseases, including diabetes, hypertension, and inflammatory bowel diseases [44,45], leading to increased permeability to gut bacteria-derived metabolites, including trimethylamine (TMA) [45]. Jaworska et al. observed increased permeability of GBB to SCFAs in an acetic acid-induced rat model of colitis, and paediatric patients with inflammatory bowel disease [44].

### 2.2. Gut Microbiota-Derived Metabolites

Several dietary nutrients were proven to undergo both endogenous and bacterial metabolism. For example, dietary choline can be absorbed in the intestines and used for the synthesis of phospholipids, vital components of cells’ membranes and lipoproteins, taking part in the transport of triglycerides and cholesterol between liver and peripheral organs [46]. Additionally, choline is used for the synthesis of acetylcholine, an important neurotransmitter of the parasympathetic nervous system regulating vital physiological functions at a resting state [47]. Furthermore, choline can also be transformed by gut microbiota, leading to the synthesis of TMA [48], which has been revealed to have a negative impact on the cardiovascular and nervous system in mammals by increasing blood pressure [49], promoting formation of atherosclerotic plaques [50] and taking part in the pathophysiology of epilepsy and behavioural disorders [51]. TMA can be then further oxidized to trimethylamine N-oxide (TMAO) by hepatic flavin monooxygenases [52]. TMAO synthesis increases after phosphatidylcholine- and L-carnitine-rich meals [53], and depends on the metabolism by gut microbiota, which was demonstrated in humans [54]. TMAO has multiple known effects on the host, from lowering blood pressure and increasing diuresis [55], to acting as an osmolyte in order to protect mammalian cells from high hydrostatic and osmotic stress [56].

Dietary substrates, e.g., sulfates, sulfites and cysteine are also transformed into hydrogen sulfide (H2S) by microbes such as *E. coli* and *Desulfovibrio* or *Enterobacter* species [57]. The vasodilatory and hypotensive properties of this gaseous transmitter have been known for decades [58,59,60]. Recently, many studies have supported the hypothesis that high and low concentrations of H2S have different biological effects on mammalian health. Namely, low levels of hydrogen sulfide seem to protect the cellular bioenergetics as well as intestinal epithelium integrity, while its high concentrations exert toxic effects [61,62].

Diet rich in fibre is associated with the growth of specific bacteria producing SCFAs, including acetic, propionic, butyric, and valeric acids [53,63]. SCFAs have a significant impact on health in mammals by showing anti-inflammatory [64], hypotensive [5,65,66] and hypolipidemic [67] effects, as well as by improving endothelial dysfunction induced by angiotensin II [68]. Moreover, SCFAs have vasodilatory properties, which were investigated in coronary and colonic circulation, among others [69,70,71,72]. Additionally, microbiota-derived indoles, metabolites of exogenous tryptophan, were proven to affect the nervous, immune, gastrointestinal, and cardiovascular systems in mammals [9,73], which will be further discussed in this review.

## 3. Tryptophan Metabolism

**Figure 1:**
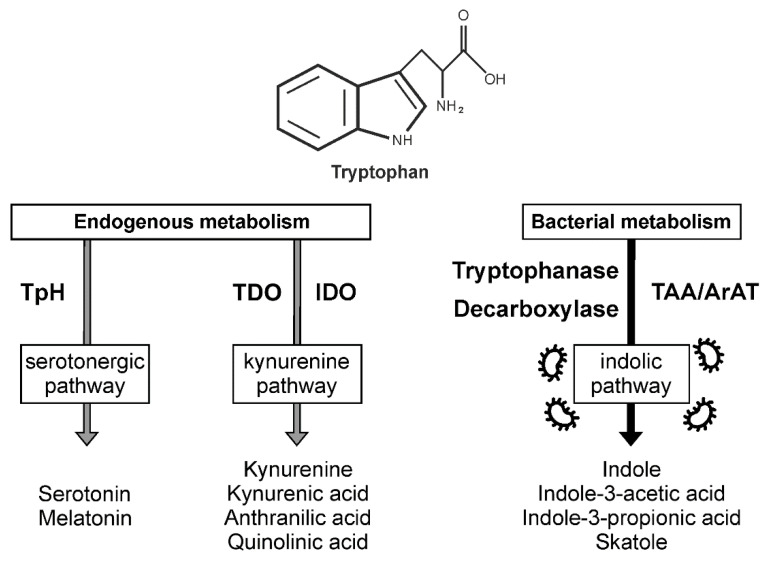
Simplified representation of metabolic pathways of tryptophan in mammals. Tryptophan can be metabolized by host’s own cells (endogenous pathways, grey arrows) and by symbiotic gut microbiota (bacterial pathways, black arrow). TpH–tryptophan hydroxylase; TDO–tryptophan 2,3-dioxygenase; IDO–indoleamine 2,3-dioxygenase; TAA–tryptophan aminotransferase; ArAT–aromatic amino acid aminotransferase.

Tryptophan is an essential amino acid vital for maintaining health and homeostasis afforded by its complex metabolism (Figure 1) and biological actions.

### 3.1. Kynurenine Pathway of Tryptophan Metabolism

In the kynurenine pathway (KP), which accounts for around 95% of tryptophan catabolism, tryptophan is oxidized to N-formylkynurenine (NFK) mainly by tryptophan 2,3-dioxygenase (TDO) located in the liver [18,74]. It is the first and rate-limiting step in this pathway and its activity is regulated by steroids, including cortisol, and systemic levels of tryptophan [75]. TDO is highly selective when it comes to substrates and works specifically with tryptophan [76]. Other enzymes, including indoleamine 2, 3-dioxygenase 1 (IDO) and indoleamine 2, 3-dioxygenase 2 (IDO2) contribute to tryptophan breakdown in extrahepatic tissues and accept other substrates as well. Under normal circumstances, those enzymes are significantly less active than TDO and thus, a great part of KP takes place in the liver. However, it has been reported that inflammation might increase the significance of extrahepatic kynurenine (Kyn) formation [75]. Furthermore, formidase transforms NFK to Kyn. It is further metabolized by numerous enzymes into its derivatives such as anthranilic acid, kynurenic acid and quinolinic acid. The latter is converted into nicotinamide adenine dinucleotide (NAD) in a final step of KP [18,74].

### 3.2. Serotonergic Pathway of Tryptophan Metabolism

Serotonergic pathway degrades only a small fraction (1–2%) of ingested tryptophan. Two essential enzymes involved in these processes are tryptophan hydroxylase 1 and 2 (TPH1 and TPH2). They produce an active metabolic intermediate, 5-hydroxytryptamine (serotonin, 5-HT), in the gut (TPH1) and in the brain (TPH2) [74], which is further transformed into melatonin. Serotonin not only works as a neurotransmitter in the central nervous system, but also controls several physiological functions from the motility of the gastrointestinal tract to glucose homeostasis [77]. Serotonin produced in the gut is released into the blood stream, where platelets use it as a signalling molecule in clot formation [78]. Its metabolites also play important physiological roles and can be used for diagnostic purposes. One of them, melatonin, regulates circadian rhythm and has anti-inflammatory properties [79]. Finally, measurement of urine levels of 5-HIAA (5-hydroxy indoleacetic acid, a waste product of serotonin breakdown), is used to estimate serotonin levels in patients with serotonin-secreting neuroendocrine tumours [80,81].

### 3.3. Bacterial Metabolism of Tryptophan

Ingested tryptophan is, in large part, absorbed in the intestines to be further metabolized by host’s cells. Fractions of this metabolite remaining in intestinal lumen can be absorbed by symbiotic microbiota, enabling bacterial growth and function. Gut bacteria use this amino acid for their own needs, simultaneously producing biologically active metabolites that can influence the host’s homeostasis. Symbiotic microorganisms directly convert tryptophan to indole, skatole, indole-3-acetic acid (IAA), IPA, and indole-3-aldehyde (IAld) [9,18,73].

#### 3.3.1. Formation of IPA by Gut Microbiota

Bacteria taking part in the gut formation of IPA include *Lactobacillus reuteri* [82], Akkermansia and Clostiridum genus [83,84], including species *Clostridium sporogenes* [85,86,87,88] and *Clostridium caloritolerans* [88], as well as some Peptostreptococci [89]. Microbial pathway of IPA production is primarily controlled by tryptophan aminotransferase (TAA, aromatic amino acid aminotransferase, ArAT) [73,89]. Additionally, it has been also established that bacterial tryptophanase enables synthesis of IPA in the gut [18]. 

#### 3.3.2. Formation of Other Indoles by Gut Microbiota

Multiple genera and species participate in the synthesis of specific indoles from tryptophan [89]. According to Roager et al. *Escherichia coli*, *Clostridium* spp. and *Bacteroides* spp. catabolize tryptophan to indole using the enzyme tryptophanase [89]. Furthermore, main bacteria producing IAA are Bacteroides such as *Bacteroides ovatus*, *B. eggerthii*, *B. thetaiotaomicron*, and *B. fragilis*, as well as some representants of the Clostridium, Bifidobacterium and Eubacterium genus [21,90]. Decarboxylase and tryptophanase take part in IAA formation [18]. Finally, several Lactobacilli can also synthesize IAld using aromatic amino acid aminotransferase [21,90].

## 4. Biological Effects of Tryptophan and IPA

### 4.1. Tryptophan and Immune System

The relationship between tryptophan and the immune system is bidirectional. On one hand, tryptophan and its metabolites have an impact on the expression of interleukins. On the other hand, it has been observed that, in the presence of inflammatory and autoimmune diseases, tryptophan metabolism shifts, leading to increased synthesis of kynurenines [91,92]. Tryptophan breakdown by IDO is associated with immune system function, since metabolites of the KP reveal immunomodulatory activity, by reducing Th-17 cells formation and promoting formation of regulatory T cells [93]. These effects justify increased IDO expression in pregnancy, as a factor enabling pregnancy tolerance in mammals [94]. Furthermore, IDO expression increases in viral [93], bacterial [95], and parasitic [96] infections and states associated with increased expression of tumor necrosis factor α (TNF-α) and interferon-γ (IFN-γ) [97]. Additionally, the expression of this enzyme is also increased in autoimmune and neurodegenerative diseases, including rheumatoid arthritis, multiple sclerosis, and Alzheimer’s disease [91]. Moreover, IDO expression increases in carcinogenesis [98] and enhanced tryptophan breakdown via KP is associated with poorer outcome and development of complications, including anaemia and fatigue, in cancer patients [99]. IDO, as a first enzyme of the KP, promotes formations of Kyn and its further metabolites, simultaneously decreasing concentration of their precursor, tryptophan. Hence, Kyn/Trp (kynurenine/tryptophan) ratio was proposed as one of the markers of increased inflammatory response [74,93]. Interestingly, tryptophan itself reveals strong antioxidative activity [100], and reduces LPS-induced lipoperoxidation [101]. Endogenous metabolites of tryptophan, including melatonin, can also act as free radicals’ scavengers [102]. Finally, decreased consumption of tryptophan in diet is associated with increased serum levels of pro-inflammatory cytokines, including IL-1alpha [103].

### 4.2. Tryptophan and Body Mass Regulation

Tryptophan is an essential amino acid and component of a balanced diet. Its presence in the diet is vital for protein synthesis, metabolism and other functions maintaining homeostasis. Research shows that excessive tryptophan consumption and tryptophan deficiency in the diet can affect body mass regulation in mammals. Rats consuming both tryptophan-low and tryptophan-free chow experienced a significant reduction in body weight gain [104,105,106]. Interestingly, supplementing tryptophan in the diet reduces food intake and weight gain in rats [105,107]. Similar effects were observed by intragastric administration of this amino acid in mice [108].

### 4.3. Tryptophan and Cardiovascular System Regulation

Regulation of the cardiovascular system involves action of many specific tissues and hormones to adapt to rapid changes in blood pressure and other hemodynamic parameters. Small molecules, such as noradrenaline and adrenaline, which are metabolites of amino acid tyrosine, are well-known regulators of cardiovascular system function [109]. Tryptophan also affects hemodynamic parameters. With tyrosine and histidine, tryptophan belongs to a group of sensitizers of β-adrenergic receptors (ESBAR) [110]. Additionally, tryptophan increases the contractility of human myocardial cells, demonstrating an inotropic property [111]. Oral administration of this amino acid increases portal blood pressure and produces a trend towards higher mean arterial blood pressure in rats [11]. Parenteral tryptophan infusion increases blood pressure in normotensive rats [112]. Additionally, tryptophan given orally and parenterally reduces sodium excretion in the kidneys revealing an antidiuretic effect [113,114]. On the other hand, parenteral infusion of tryptophan decreases blood pressure in spontaneously hypertensive rats [112]. The hypotensive effect was also observed after oral administration to patients with essential hypertension [115]. Moreover, IDO activity is also associated with blood pressure regulation. Increased IDO activity in mice infected with malarial parasite was associated with a decrease in systolic blood pressure. Interestingly, inhibition of IDO significantly increased blood pressure in infected mice [96], a pattern similar to effects of tryptophan administration in normotensive rats [112]. These observations show complex biological effects of tryptophan that might be explained, at least partially, by the action of its endogenous and microbiota-derived metabolites, including IPA.

## 5. Biological Effects of IPA and Its Impact on Health in Mammals

**Figure 2:**
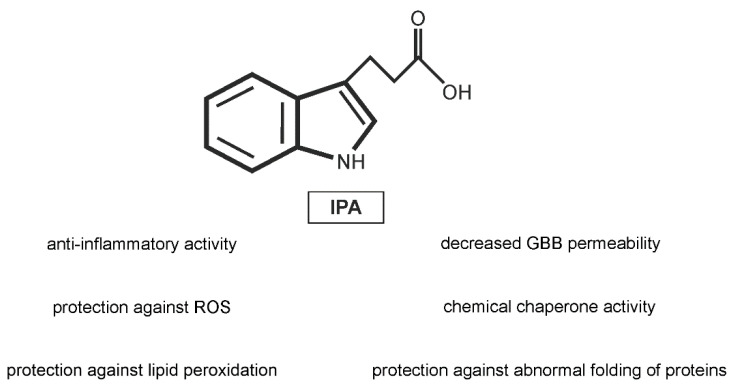
Protective effects of indole-3-propionic acid (IPA) on cellular and tissue level. GBB- gut-blood barrier; ROS–reactive oxygen species.

Knowledge on the biological action of IPA has increased significantly over the past several years, giving new evidence on the positive and protective effects of this metabolite (Figure 2).

### 5.1. IPA Improves Gut-Blood Barrier Function

Studies on the GBB demonstrate that indole [10] and IPA [20,116,117], two tryptophan metabolites, improve barrier properties by increasing the expression of claudins and other tight junction proteins. Additionally, IPA increases secretion of mucins in in vitro human colonic culture [20] and increases the number of goblet cells and mucosa thickness in rats [118]. Moreover, IPA acts as a ligand of the aryl hydrocarbon receptor (AhR) present in colonic epithelial cells, activation of which is associated with anti-inflammatory and anticancer effects [119] Interestingly, it has been observed that in patients suffering from colitis serum, IPA concentration decreases significantly [28]. Apart from being an AhR ligand, IPA can also produce its biological action by activating the pregnane X receptor (PXR) present in colonic, liver endothelial and muscle cells [23,119,120]. Complex laboratory techniques allow testing the function of GBB by measuring its permeability to specific substances, including Fluorescein Isothiocyanate-Dextran (FITC-Dextran). Three independent research projects revealed that IPA decreases FITC-dextran-dependent gut permeability in mice [121,122,123]. Venkatesh et al. proved that IPA decreases gut permeability by interacting with PXR [121]. 

### 5.2. IPA Protects against Oxidative Stress and Attenuates Inflammation

Physiological activity of the immune system is important in the regulation of mammalian health and represents a dualistic approach. Its activation limits the invasion of pathogens and is a key mechanism protecting from infections. On the other hand, regulatory mechanisms moderating the immune response must be preserved to prevent the excessive activation of immune cells associated with autoimmune diseases. Bacterial metabolites of tryptophan have a complex impact on the immune system, revealing both pro- and anti-inflammatory properties. IS, the liver metabolite of microbiota-derived indole, promotes the production of reactive oxygen species (ROS) [124] and induces expression of proinflammatory cytokines, including IL-1β [125], TNF-α [125] and MCP-1 [125,126,127]. Interestingly, IPA protects cells from ROS [25,128,129], oxidative damage and lipid peroxidation caused by potassium bromate [130,131,132], potassium iodate [24], iron (II) sulphate [133,134], iron (III) chloride [135], chromium (III) chloride [136], and 2,2′-Azino-bis(3-ethylbenzothiazoline-6-sulfonic acid (ABTS) [137]. Additionally, IPA decreases the expression of proinflammatory cytokines, including TNF-α [22,27,121], IL-1β [23,27], IL-6 [27], IL-12 [22], IL-13 [22] and MCP-1 [22,82]. Interestingly, IPA also shows antimicrobial activity by inhibiting the growth of *Legionella pneumophila* [138,139], and *Salmonella typhimurium* [140]. Apart from that, in patients with type II diabetes, serum concentration of IPA is negatively correlated with high-sensitivity C-reactive protein (hsCRP) [141]. Moreover, Nyström et al. observed that in immunodeficient patients with HIV infection synthesis of IPA is significantly diminished [142].

### 5.3. IPA Protects against Carcinogens and Has an Antitumor Potential

Carcinogenesis is a complex and complicated process of modification of healthy cells into autonomous and self-sufficient pathogenic conglomerate of cells. Many factors enhancing carcinogenesis have been established, including ionizing radiation, ultraviolet (UV) radiation, oxidative stress, DNA damage, viral infections, and lifestyle factors, such as tobacco smoke, ethanol consumption, and ingestion of nutritional carcinogens [143,144]. Increased formation of ROS has a negative impact on lipid barriers, affects DNA structure, and takes part in carcinogenesis; therefore antioxidants, both nutritional and pharmacological, have been targets of great research interest over the past years [145,146]. Both melatonin and IPA can be classified as free radical’s scavengers, and their antitumor potential have been investigated in the scientific literature [136,147]. IPA prevents DNA damage in hamsters’ kidneys exposed to oestradiol [147], rats’ brains exposed to chlorpyrifos [148], and calf thymus samples exposed to chromium (III) chloride [136]. Additionally, IPA decreases fluidity of rats’ liver microsomal membranes incubated with chromium (III) chloride [149]. Due to its positive cellular effects, IPA has become a promising particle used for the chemical modification of well-known antineoplastic drugs [150] and compounds [151,152]. Addition of an IPA particle as a ligand to a cisplatin structure caused significant cytotoxic effect, associated with increased ROS formation [150]. Cadmium-IPA complexes possess antiproliferative and proteasome-inhibitory activity in in vitro breast cancer cells [151]. Interestingly, IPA alleviates hematopoietic and gastrointestinal side effects of radiotherapy in the mice model [153]. Further research projects should focus on testing the impact of IPA on neoplastic cells’ survival and evaluate its role in both cancer prevention and therapy. 

### 5.4. IPA Has a Protective Role in Neurodegenerative Disease Models 

The pathophysiology of neurodegenerative diseases is complex, and many possible pathways have been proposed as therapeutic targets to slow down disease progression and preserve neurological functions of the affected individuals. In general, the development of Alzheimer’s, Parkinson’s and Huntington’s diseases is associated with pathological accumulation of specific proteins in vital brain centres responsible for cognition, memory, motor activity and other essential neurological processes [154,155].

#### 5.4.1. IPA in Alzheimer’s Disease

Abnormal folding of amyloid β-proteins and their deposition in amyloid plaques reveals neurodegenerative properties due to the activation of ROS [156]. 

Melatonin has antioxidative action, and also increases clearance of amyloid β-protein in the mice model of Alzheimer’s disease amyloidosis [157]. IPA shares protective biological effects against oxidative stress with melatonin [24,133] and decreases aggregation of amyloid β-protein in in vitro experiments [158]. The addition of IPA to media of the primary neurons and neuroblastoma cells exposed to amyloid β-protein significantly reduced toxicity and prevented cell death [159]. Dragicevic et al. observed that both IPA and melatonin restore mitochondrial function in the in vitro model of Alzheimer’s disease [160]. Interestingly, IPA also protects brain tissue from oxidative stress, lipid peroxidation and oxidative DNA damage in hippocampal region after acute brain ischemia [26]. Additionally, IPA acts synergistically with glutathione to prevent ABTS-related formation of free radicals in the rat brain and reduces associated lipid peroxidation [128]. Surprisingly, Huangs et al. observed a trend towards higher plasma IPA levels in patients with progressive mild cognitive impairment (MCI) and Alzheimer’s disease, compared to patients with stable MCI. However, these changes were not statistically significant [161]. Additional research data are needed to fully evaluate role of IPA as a protective or predictive factor in Alzheimer’s disease.

#### 5.4.2. IPA and Other Neurodegenerative Diseases

IPA exerts a chemical chaperone-like activity and inhibits abnormal aggregation of the regular [162] and denatured [163] proteins. In two cell culture models of Parkinson’s disease associated with overexpression of Parkin-associated endothelin receptor-like receptor (Pael-R) and α-synuclein, IPA significantly reduced ROS-associated cell death [163] Additionally, patients with Huntington’s disease have lower plasma concentration of IPA, which might be associated with system-wide decreased ability to protect against ROS formation [87]. On the contrary, increased IPA formation was observed in mice with experimental autoimmune encephalitis (EAE) [164], animal model of multiple sclerosis (MS), and patients with relapsing-remitting MS [165].

### 5.5. IPA Has a Positive Impact on Cardiovascular Disease Risk Factors

Initially endogenous metabolites of tryptophan were suspected to be accountable for tryptophan’s cardiovascular actions [166]. Recently, it has been established that IPA itself can also affect cardiovascular system [19,167]. Additionally, its synthesis can change in presence of cardiovascular-related diseases (Table 1).

#### 5.5.1. Diet

The composition of specific nutrients in diet can affect mammalian health and disrupt lipid and carbohydrate homeostasis. Diets rich in red meat and saturated fatty acids are long-established cardiovascular disease risk factors [171]. Additionally, diets with a high content of red meat increase the synthesis of TMA, a bacterial metabolite formed mainly from carnitine [6,172]. Complex nutritional interventions stimulate the formation of other microbiota-derived metabolites, including IPA. The Mediterranean diet, known for its beneficial and cardio-protective role, significantly increases the plasma concentration of IPA in humans [168]. Moreover, diets rich in fibre [29,141] and inulin [173] also promote the synthesis of this metabolite. On the contrary, a fast-food diet, a known risk factor for cardiovascular diseases, significantly reduces the plasma concentration of IPA in humans [168]. Interestingly, the addition of IPA to a high-fat diet in mice restores bone mineralization and osteoblasts’ function diminished in mice ingesting a high-fat diet alone [174].

#### 5.5.2. Dyslipidaemia

Disturbances in lipid metabolism, including the rise in total cholesterol levels, have been linked to an increased risk of cardiovascular disease-related mortality [175]. Association of high concentration of triglycerides and low HDL cholesterol level is classified as atherogenic dyslipidaemia and is linked to the progression of atherosclerosis [176]. Long-lasting disturbances in lipid metabolism lead to obesity, metabolic syndrome and non-alcoholic fatty liver disease [177]. Plasma concentration of IPA is significantly reduced in patients with dyslipidaemia [169]. Hypolipidemic interventions, including the administration of mulberry leaf extract and 1-deoxynojirimycin, are associated with increased IPA concentration in stools [83]. Additionally, IPA plasma concentration correlates negatively with lipid parameters, including triglycerides and LDL-C plasma levels [173]. Moreover, IPA reduces hepatic steatosis and hepatocyte dysfunction in a rat model of high-fat diet-induced steatohepatitis [27]. In patients with hepatic lobular inflammation and liver fibrosis significant decrease in circulating IPA levels in serum can be observed [178]. Surprisingly, IPA enhances liver damage in mice with carbon tetrachloride-induced liver fibrosis, without affecting the liver functions of healthy controls [179]. Previous observations need to be further investigated to establish whether IPA has a positive or negative effect on liver function. There is only one research paper, in which IPA failed to reveal protective metabolic effects in mice fed Western diet, while simultaneously improving intestinal functions [122]. Taken together, more research data is needed to fully understand regulatory role of IPA in lipid, metabolic, and liver homeostasis. It is possible that specific metabolic changes occur when the concentration of this metabolite in peripheral blood increases above a certain threshold that needs to be established in further experiments.

#### 5.5.3. Obesity

The number of patients with excess body fat increases drastically each year, and data extrapolations suggest this trend will continue [180]. Increased body weight and obesity are associated with a broad group of metabolic disturbances due to excessive adipose tissue accumulation and increased cytokine synthesis [181]. Previously, we discussed the positive role of tryptophan in the reduction in body mass. Recent results from our laboratory showed that parenteral administration of IPA significantly recuses weight gain in rats [105]. Additionally, human studies showed that in obese female patients, a significant decrease in the concentration of IPA in serum and follicular fluid could be observed compared to women with normal weight [30]. Apart from that, in an animal model of glucocorticoid withdrawal syndrome, IPA formation is significantly diminished, showing that its synthesis might also by affected by changes in the function of the adrenal glands [182].

#### 5.5.4. Hyperglycaemia

Increased plasma glucose concentration is associated with severe complications, including endothelial dysfunction, atherosclerosis progression, and lipid homeostasis changes [183]. Diabetes and prediabetic states are considered significant risk factors for cardiovascular disease, the prime cause of death in this patient population [184]. Despite multiple therapeutic options and new innovative treatments, many patients struggle to control their glycaemia adequately. New research findings suggest that carbohydrate homeostasis might be affected by metabolites produced by gut bacteria, including indoles [29,185]. Abildgaard et al. observed that introducing an IPA-enriched diet for 6 weeks significantly reduces fasting blood glucose concentration and plasma insulin level in rats [186]. Furthermore, in a human study of the Finnish population, patients who developed type II diabetes had significantly lower serum concentration of IPA [29]. Additionally, the concentration of this metabolite has been inversely correlated with the incidence of type II diabetes and tended to be positively correlated with insulin secretion [141].

#### 5.5.5. Hypertension

Blood pressure control depends on two main components: mechanical action of the myocardium and peripheral vasculature [187]. Elevated blood pressure is an important cardiovascular risk factor, as it takes part in the pathophysiology of atherosclerosis and causes microcirculatory dysfunction and progressive tissue damage [188]. Bacterial metabolites of tryptophan reveal similar hemodynamic patterns as their precursor. Results from our laboratory show that both IS and indole increase blood pressure in normotensive rats, with and without a concomitant increase in heart rate [13]. The scientific literature indicates that IPA is also involved in blood pressure regulation in mammals [19]. Using the Langendorff heart model in mice, an IPA-dependent increase in myocardial contractility was demonstrated [167]. Additionally, this metabolite causes vasoconstriction of the endothelium-denuded mesenteric resistance arteries [19] and diminishes vasodilatation associated with pre-treatment with sodium nitroprusside [189] and acetylcholine [19]. The vascular effect of IPA might be mediated by the activation of PXR [189]. So far, it has not been elucidated whether long-term administration of IPA takes part in the pathophysiology of hypertension and hypertension-related cardiovascular diseases. Human studies revealed that patients with advanced atherosclerosis had significantly reduced plasma IPA concentrations [170]. Additionally, in this group, IPA levels correlated strongly with higher ankle-brachial index (ABI) and less severe peripheral arterial disease (PAD) [170].

## 6. Modulation of IPA Concentration as a Therapeutic Target

### 6.1. Antibiotics

Knowledge of antibiotics and their potential in treating infections dates back to the discovery of penicillin by Alexander Flemming in 1928 [190]. Nowadays, antibiotics are some of the most frequently prescribed medications in everyday medical practice. Growing evidence suggests that using antibiotics affects pathogenic bacteria and strikes back against symbiotic microbiota, leading to the development of serious diseases, including *Clostridioides difficile* infection and pseudomembranous colitis [191]. Antibiotic groups differ in their pharmacokinetic properties, which affects their distribution and action site in the organism. For example, neomycin given orally acts predominantly in the gut due to its low absorption in the intestines [192]. Behr et al. tested how oral administration of specific antibiotics affects the synthesis of certain bacterial metabolites, including IPA. A 4-week treatment with fluoroquinolones, tetracyclines and aminoglycosides significantly reduced IPA plasma concentration, with the greatest effect on the third group [193]. Data from our laboratory confirmed the observation, as mentioned above. A 2-week-long oral administration of neomycin significantly reduced the levels of IPA in the stool, portal, and peripheral blood of Sprague Dawley rats [105]. Other antibiotics, including ampicillin, might also affect the bacterial metabolism of tryptophan [82].

### 6.2. Tryptophan Concentration in Diet

Several factors affect IPA metabolism in mammals. Diet, medications, and intestinal disturbances can influence the concentration of this metabolite in the gut. The introduction of a tryptophan-rich diet for 2 weeks significantly increases the concentration of IPA in colon contents, portal, and peripheral blood [105]. On the other hand, a tryptophan-free diet, administered for the same period, significantly reduces the synthesis of this metabolite [105]. Tryptophan concentration in the diet is not the only nutritional factor affecting the synthesis of IPA [83,168].

### 6.3. Probiotics

Administration of specific bacterial species as probiotics has increased its recognition as a therapeutic option in gastrointestinal diseases over the past few years [194,195]. Probiotics co-administered with antibiotics seem to decrease the risk of developing severe adverse effects, including *Clostridioides difficile* infection [194]. Probiotics restore healthy gut microbiota composition and its functions, including synthesis of specific metabolites [174,196,197,198]. *Lactobacillus reuteri* can be administered as a probiotic, revealing a positive impact, by improving infantile colic symptoms [199,200]. Simultaneously it has been observed that this bacterial species is able to synthetize IPA [82]. It still needs to be elucidated whether the positive impact of *L. reuteri* might be due to the synthesis of specific metabolites, including IPA. 

## 7. Materials and Methods

**Figure 3:**
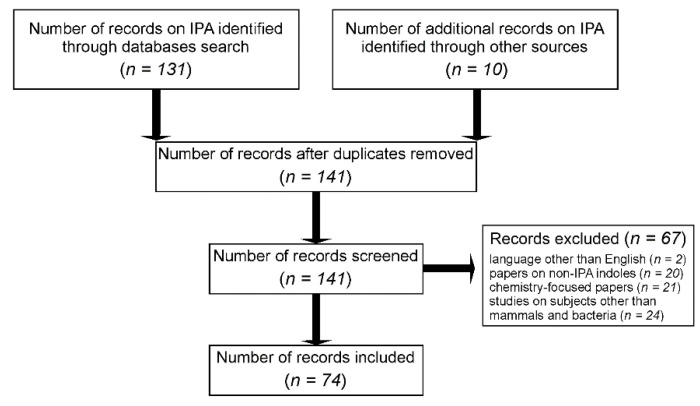
Schematic representation of study protocol of papers evaluating synthesis and biological effects of IPA. Black arrows show steps of the review process. IPA–indole-3-propionic acid.

We searched PUBMED and Google Scholar databases to identify pre-clinical and clinical studies on synthesis and biological effects of IPA. The key words included microbiota, tryptophan, indoles, indole-3-propionic acid. The search was confined to manuscripts that were published from 1961 to December 2021. Specific steps of the review process and the evaluation of scientific papers are present in Figure 3. A total of 131 records on IPA were obtained from databases, and an additional 10 papers were included from other sources. A total of 141 papers were screened for relevance and 67 records were excluded from further analysis. Exclusion criteria were language other than English (2 records), chemistry-focused papers (21 records), papers evaluating results from experiments on subjects other than mammals and bacteria (24 records), and papers on indoles other than IPA and not evaluating effects of IPA (20 records). Finally, 74 papers evaluating the synthesis and biological effects of IPA were included in this review.

## 8. Conclusions

Symbiotic gut microbiota is able to produce biologically active metabolites that can affect functions of the host. IPA belongs to the wide group of indoles, microbiota derived metabolites of tryptophan. It has been observed that IPA has beneficial impact on host health by possessing anti-inflammatory and ROS-scavenging activity. Further research projects should evaluate its possible clinical applications in the treatment of autoimmune, inflammatory and oncological diseases. Additionally, its synthesis decreases in many pathological states associated with an increased risk of developing cardiovascular diseases, making it a promising new pharmacotherapeutic target.

## Figures and Tables

**Table 1:** Comparison of tendencies in the synthesis of indole-3-propionic acid (IPA) associated with protective and harmful factors of cardiovascular diseases.

Impact of a Factor on Cardiovascular Health	Factor Affecting Cardiovascular Health	Change in the Synthesis of IPA	References
Positive	Mediterranean diet	Increase	[168]
	Increased composition of fibre in the diet	Increase	[29,141]
	Increased mulberry consumption	Increase	[83]
Negative	Diabetes	Decrease	[29]
	Dyslipidaemia	Decrease	[169]
	Obesity	Decrease	[30]
	Atherosclerosis	Decrease	[170]

## Data Availability

All relevant data are presented in this paper.
